# Iris Pigmented Lesions and Risk of Cutaneous Melanoma: Case–Control Study in Lithuania

**DOI:** 10.3390/jpm14050530

**Published:** 2024-05-15

**Authors:** Lukas Šemeklis, Laura Kapitanovaitė, Grinvydas Butrimas, Kamilija Briedė, Augustė Dubinskaitė, Reda Žemaitienė, Skaidra Valiukevičienė

**Affiliations:** 1 Department of Ophthalmology, Medical Academy, Lithuanian University of Health Sciences, LT-44307 Kaunas, Lithuania; 2 Department of Ophthalmology, Hospital of Lithuanian University of Health Sciences Kauno Klinikos, LT-50161 Kaunas, Lithuania; 3 Department of Skin and Venereal Diseases, Medical Academy, Lithuanian University of Health Sciences, LT-44307 Kaunas, Lithuania; 4 Department of Skin and Venereal Diseases, Hospital of Lithuanian University of Health Sciences Kauno Klinikos, LT-50161 Kaunas, Lithuania; 5 Faculty of Medicine, Medical Academy, Lithuanian University of Health Sciences, LT-44307 Kaunas, Lithuania

**Keywords:** cutaneous melanoma, iris pigmented lesions, iris patterns, iris freckles, cutaneous melanoma

## Abstract

The global incidence of cutaneous melanoma (CM) is rising, necessitating early detection and identification of risk factors across different populations. A case–control study with 180 patients with primary diagnosed CM and 182 healthy controls was conducted. Participants underwent ophthalmic and skin examinations, where the identification and counting of common melanocytic nevi (CMN) and atypical melanocytic nevi (AMN) was performed. During ophthalmic examination, high-resolution slit lamp iris images were taken. Images were categorized according to iris periphery, collaret, and freckles. There was no difference in iris periphery and collaret color between groups. However, blue/grey iris periphery and blue collaret with or without freckles were the most common patterns. The presence of pigmented iris lesions and 2–5 mm and ≥5 mm in diameter CMNs was strongly associated with CM risk. The evidence from this study indicates that blue or grey periphery and blue collaret iris pattern with iris freckles are 2.74 times higher in the CM group than controls. Further research is needed to explore iris patterns’ association with CM risk in diverse populations.

## 1. Introduction

Cutaneous melanoma (CM) is a malignant skin cancer that originates in melanocytes, the cells responsible for producing the pigment melanin. CM is known for its potential to spread rapidly either by the lymphatic or the hematogenous route, making early detection crucial for successful treatment [1].

The incidence of CM has been on the rise globally [2,3]. In the last few decades the incidence and mortality rates of CM have increased, and estimated rates will increase up to 50% by 2040 [4,5]. Between 1991 and 2015, the overall CM rates in Lithuania increased by an annual percent change of 3.9% in men and 2.3% in women; correspondingly, the overall number of CM deaths increased from 64 to 103 deaths per year, and the age-standardized rate increased by 1.3 times [5].

Light hair, light eyes and fair skin color, skin type I and II according to Fitzpatrick, the number of common melanocytic nevi (CMN) and the presence of atypical melanocytic nevi (AMN) and freckles, a higher level of education, and a history of CM melanoma are considered as risk factors for CM. Exposure to UV radiation, especially through sunburn during childhood, as a risk factor for CM remains debatable [6,7,8].

A recent study has shown a greater number of conjunctival naevi, iris naevi, iris freckles and choroidal naevi in patients with dysplastic naevus syndrome than in a healthy population [9]. Besides the presence of AMN, iris naevi were considered a marker of a predisposed phenotype at risk of uveal melanoma [10]. Light eye color has been considered a predictive factor for CM [11,12]. Iris freckles are a potential biomarker of chronic sunburn damage [13]. Moreover, blue iris periphery dark collaret and iris freckle patterns have been proven as reliable phenotypic markers for epidermal skin cancer in the Southern Europe population [14]. In addition, iris pigmented lesions and their count provide additional predictive information for cutaneous melanoma risk [15].

Risk factors for ocular melanoma are similar to those for CM, and include light eye color and fair skin. A family history of ocular melanoma, dysplastic nevi syndrome, xeroderma pigmentosum, and AMN also have increased risk of ocular melanoma. There is a significant overlap in patient populations with ocular melanoma and CM. Therefore, not only should CM risk factors be considered as uveal melanoma risk factors but also vice versa. Accordingly, the present study attempts to investigate pigmentary iris lesions and iris patterns as risk factors for CM. Although, until now, no data have been published about CM risk factors in the Baltic countries, this is the first study on CM risk factors, as well as on the association between CM and pigmented iris lesions.

## 2. Materials and Methods

### 2.1. Study Design

A hospital-based case–control study was performed at the Hospital of Lithuanian University of Health Sciences Kauno Klinikos. The case group was formed from patients with primary diagnosed CM (*n* = 180). The CM diagnosis was based on a histopathologic report and the decision of multidisciplinary melanoma teams in the study center. The control group (*n* = 182) was composed of dermatologically healthy participants. We invited patients with primary CM diagnosed from 2017 to 2023 to participate. Subjects’ invitation, ophthalmological and dermatological examination, and data collection took place from 2022 to 2024.

All eligible patients with CM were invited to participate in the study. After recruitment to the case group, an equivalent by age and sex control group was formed.

The study was conducted according to the principles of the Declaration of Helsinki and approved by the Kaunas Regional Biomedical Research Ethics Committee (2021-06-09 No. BE-2-66). All participants in the study have signed a written informed consent.

### 2.2. Clinical Examination

**Figure 1:**
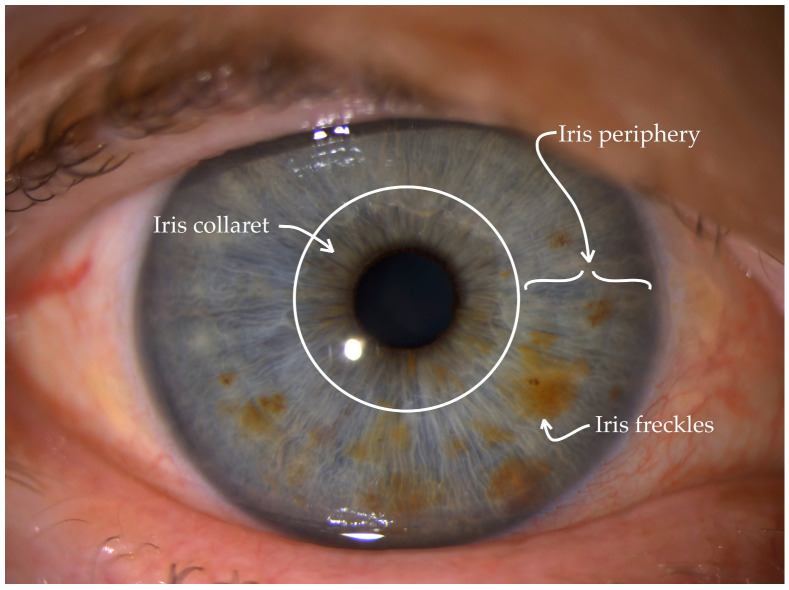
Example of iris image with explanation of iris periphery, collaret, and iris freckles evaluation.

All subjects underwent ophthalmic and skin examinations. The ophthalmic examination consisted of best-corrected visual acuity measurement, tonometry, and slit lamp examination. Additionally, high-resolution slit lamp iris images of both eyes were taken with a BQ 900 (Haag-Streit) slit lamp and IL 900 (Haag-Streit) imaging module. All iris images were taken with standardized shadow-free settings—16× magnification, opened and diffused slit illumination with 45° angle, slit illumination level—4, background illumination level—3, and focus on iris. Iris images were analyzed and classified according to the Descriptive Iris Color Classification Scale [16]. An example of an iris image is shown in Figure 1.

The Descriptive Iris Color Scale evaluates three iris parameters: iris concentric periphery color, collaret–iris central peripupillary color and the presence of iris freckles. Iris periphery was evaluated as 1 if color was blue/grey, 2 if green, 3 if hazel, 4 if light brown, and 5 if dark brown. Collaret was evaluated as 1 if color was blue, 2 if light brown and 3 if dark brown. The presence of iris freckles was evaluated as 1, and the absence of iris freckles was 0. Depending on the assigned digits, a three-digit number of the iris pattern was compiled. Both eyes’ encoded iris images were analyzed by two ophthalmologists separately. No periphery and collaret color differences between eyes were found. Only a few differences in iris freckles between left and right irises were found. In these cases, the presence of iris freckles was recorded into the final iris pattern classification. Low-quality or out-of-focus iris images were excluded from the study. The number of pigmented lesions in each iris was counted, and the average of these lesions including each iris was calculated.

For skin examination, a standardized protocol was defined for the counting and identification of CMN [17]. Melanocytic nevi were defined as brown to black pigmented macules or papules of any size, darker in color than the surrounding skin, excluding lesions with the clinical characteristics of freckles, solar lentigines, or café au lait spots. No attempt was made to differentiate lentigo simplex from junctional MN. Skin-colored palpable lesions with the morphological features of compound or dermal CMN, halo nevi, nevi spili, congenital nevi, blue nevi, and AMN were numbered separately but were included in the total number of CMN. The body surface was divided into 26 subsites, excluding buttocks and genitalia. A metric mole analyzer with circles ranging from 1 to 12 mm was used to measure CMN of any size. Melanocytic nevi were measured without stretching the skin, and the size was assessed if the greatest dimension of the lesion touched both sides of the circles. CMN were categorized to groups from 2 mm to 5 mm or 5 mm and larger according to the diameter of CMN. AMN was defined according to the clinical criteria of the ABCDE rule (asymmetry, border irregularity, mixed color, diameter ≥ 5 mm, and erythema at the margins or elevation of the lesions). The diagnosis of an AMN was established if at least 3 of the 5 clinical criteria were fulfilled. Epiluminescent microscopy was used to further differentiate AMN from other pigmented lesions. However, AMNs were not analyzed separately. We assessed the diagnostic validity of CMN and/or AMN evaluation of one of the authors (S.V.) in a pilot sub-study. Skin examination was performed by 3 medical doctors trained by an experienced dermatologist (S.V). The clinical judgment regarding CMN and AMN differed by less than 5% from the judgment of an experienced dermatologist (S.V.). Hair, skin, and eye color were recorded. Skin color was estimated using a 12-skin-tone panel for the left buttock.

### 2.3. Data Analysis

Data entry was performed using Microsoft Office Excel. Data analysis was conducted using the 29.0 version of IBM SPSS Statistics software.

Quantitative variables were described by providing the mean and standard deviation (SD) or the median with the lowest and highest values. Qualitative variables were described by presenting the frequency and relative frequency in the comparative samples.

The distribution and homogeneity of variance were checked before applying parameter tests. Quantitative variables between two groups were compared using Student’s *t*/Mann–Whitney tests. Qualitative variables between groups were compared using Chi-square/Fisher’s exact tests.

Multivariable binary logistic regression was performed. An age- and sex-adjusted odds ratio (AOR) was used to determine the association between the variables with a statistically significant level at a 95% confidence interval (CI). Differences were considered statistically significant with a statistical significance level < 5% (*p* < 0.05).

## 3. Results

The mean age of subjects in the case group was 59.56 years, while in the control group, it was 57.58. Both groups were distributed by age and sex equally (Table 1). The observation of the larger proportion of the female sex corresponded to the proportion of the female sex recorded in Lithuanian health statistical database among those with CM [18]. The highest proportion of subjects was over the age of 60, reaching 50.6% in the case group, and 50.5% in the control group. While analyzing the iris images, we found that there was no difference in iris periphery and collaret color between the case and control groups. However, we found that the presence of pigmented iris lesions and the average number of iris lesions was higher in the case group than between controls. The number of 2–5 mm and larger than 5 mm CMNs was higher in the case group than between controls. Other characteristics of investigated CM risk factors are shown in (Table 1).

**Figure 2:**
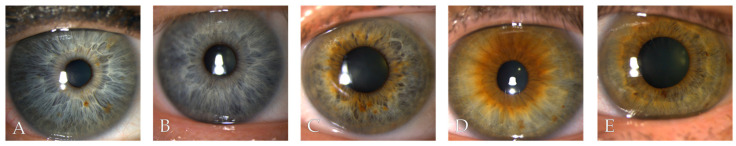
Most found iris patterns categorized according to the Descriptive Iris Color Classification Scale [16]. (**A**) Iris pattern with blue/grey periphery and blue collaret iris with freckles. (**B**) Iris pattern with blue/grey periphery and blue collaret iris without freckles. (**C**) Iris pattern with blue/grey periphery and light brown collaret iris with freckles. (**D**) Iris pattern with hazel periphery and light brown collaret iris with freckles. (**E**) Iris pattern with green periphery and light brown collaret iris with freckles.

Analysis of iris patterns showed that the most common iris patterns were with blue/grey iris periphery and blue collaret with or without freckles. In both groups, irises with blue/grey periphery and blue collaret accounted for more than half of the cases. Iris pattern “111” was more often found in patients with cutaneous melanoma, and the pattern “110” was more often found in healthy individuals. Our study identified a total of 20 distinct iris patterns. The five most found iris patterns are shown in Figure 2. The most popular iris patterns among our population were 111, 110, 121, 321, 221, as categorized according to the Descriptive Iris Color Classification Scale [16]. Comparison of the most found iris patterns among subjects with cutaneous melanoma (case group) and controls is demonstrated in Table 2.

Multivariable binary logistic regression showed that the odds of CM is higher in subjects with the presence of pigmented iris lesions (AOR = 18.75; 95% CI: 8.99; 39.12) and an average number of pigmented iris lesions (AOR = 1.13; 95% CI: 1.06; 1.21). The data revealed that the risk of CM is lower in subjects with medium skin color (AOR = 0.26; 95% CI: 0.11; 0.62) or with skin type II (AOR = 0.50; 95% CI: 0.26; 0.95) compared with the corresponding groups. Analysis of the five most frequent iris patterns (Table 2) revealed that subjects with iris pattern 111 have a higher risk of CM (AOR = 2.74, 95% CI: 1.76; 4.27). Other regression models with the most frequent iris patterns were considered as not significant. Table 3 presents the results from multivariable binary logistic regression.

## 4. Discussion

The first study of CM risk factors, including constitutional factors, the number of CMN, and iris pigmented lesions of investigated subjects in Northern Europe countries was performed. One of the more significant findings to emerge from this study is that the presence of pigmented iris lesions and a median number of CMN 2–5 mm and ≥5 mm in diameter are strongly associated with the risk of CM. Our study results suggest that blue or grey periphery and blue collaret iris patterns with iris freckles are 2.74 times higher in the CM group than in controls.

These findings are consistent with the findings of other studies by Grigore et al., which revealed that for the South-Eastern European population, blue periphery and light brown collaret and iris freckle pattern is a reliable phenotypic marker for epidermal skin cancer [14]. However, we found that blue or grey periphery and blue collaret iris pattern with or without iris freckles is more often found between patients with CM. In addition, our study showed that patients with CM had pigmented iris lesions less often than in the study performed in South-Eastern Europe.

Research findings by Laino et al. also point towards pigmented iris lesions and a higher CM risk in the Australian population. The authors showed that the number of iris pigmented lesions was associated with CM risk even after adjusting for known predisposing host factors such as skin and eye colour, skin freckling and naevi count [15].

However, there are only a few studies that assess CM risk regarding different iris patterns and iris freckles [14,15]. The iris patterns associated with CM differ slightly between different geographical populations. These differences between results could be determined by different eye, hair and skin color distribution.

Eye color distribution among European countries has revealed interesting patterns, highlighting the diversity within the continent. Northern European countries tend to have a higher prevalence of light eye colors, such as blue and green, compared to Southern European countries [19]. The frequency of light iris colors decreases from northwest to southeast in Europe. Individuals with blue, grey, or green/hazel eyes may have a higher susceptibility, possibly due to a combination of genetic factors and reduced melanin protection in the eyes [20]. So far, the complex genetics underlying eye color diversity in European populations is not clear and fully understood.

The strengths of this study conclude that the collaborative effort of dermatologists and ophthalmologists on iris pigmented lesions evaluation and skin examination underscores its interdisciplinary approach, enhancing the depth and reliability of the findings. Utilizing slit lamp cameras specifically designed for high-quality iris imaging further solidifies the credibility of the data collected. Notably, this is the first study in North and Eastern Europe evaluating the relationship between iris pigmented lesions and CM, thus filling a critical gap in the existing literature.

However, it is important to acknowledge the limitations and challenges inherent in this study. Notably, data of sun exposure were not analyzed in this study, and this may be considered as one of the limitations. Sun exposure’s association with pigmentary iris changes and CM risk could be the subject of further study. Another considered limitation of this study could be the testing of genetic markers. The scope of this study did not include genetic testing. However, testing genetic markers associated with CM and iris pigmentary changes remains a focus for future investigations. One of the challenges faced during the recruitment period was maintaining sex proportions within the control group. Despite encountering a higher proportion of females in the case group recruitment, aligning with demographic records of cutaneous melanoma (CM) cases in the Lithuanian health statistical database, achieving equivalent age and sex groups in the control group presented minor difficulties. Future research may benefit from incorporating this variable to provide a more comprehensive understanding of the relationship between iris pigmented lesions and CM risk. Despite the vast difference in eye, hair, and skin color between different populations, recent studies agree that in general, light color (blue and grey) patterns with iris freckles are risk factors for cutaneous melanoma.

## Figures and Tables

**Table 1:** Characteristics of study subjects among cutaneous melanoma case group and controls.

Characteristics	Control Group *n* = 182	Case Group *n* = 180	*p*-Value
Sex, % (*n* ^1^)			0.892
Male	37.9 (69)	37.2 (67)	
Female	62.1 (113)	62.8 (113)	
Total	182	180	
Age (years), mean (SD ^2^)			
Male	57.97 (8.20)	60.70 (13.50)	0.155
Female	57.34 (7.83)	58.88 (11.89)	0.249
Total	57.58 (7.95)	59.56 (12.51)	0.072
Age groups (years), % (*n*)			
≤50	23.6 (43)	22.8 (41)	
51–60	25.8 (47)	26.7 (48)	0.974
≥61	50.5 (92)	50.6 (91)	
Skin color, % (*n*)			
Fair	29.6 (52)	48.6 (87)	
Medium	65.9 (116)	40.8 (73)	<0.001
Olive	4.6 (8)	10.61 (19)	
Skin type (Fitzpatrick scale), % (*n*)			
Type I	15.3 (27)	21.7 (39)	
Type II	39.5 (70)	36.1 (65)	0.061
Type III	30.5 (54)	21.1 (38)	
Type IV	14.7 (26)	21.1 (38)	
Hair color, % (*n*)			
Light brown	51.7 (91)	62.0 (111)	
Dark brown	33.5 (59)	25.1 (45)	0.134
Black	14.8 (26)	12.8 (23)	
Iris periphery color, % (*n*)			
Blue/grey	70.5 (124)	70.1 (122)	
Green	14.2 (25)	13.2 (23)	
Hazel	6.3 (11)	8.6 (15)	0.730
Light brown	6.3 (11)	6.9 (12)	
Dark brown	2.8 (5)	1.2 (2)	
Iris collaret color, % (*n*)			
Blue	63.1 (111)	62.6 (109)	
Light brown	31.8 (56)	28.7 (50)	0.397
Dark brown	5.1 (9)	8.6 (15)	
Presence of pigmented iris lesions, % (*n*)			<0.001
Yes	48.9 (86)	94.8 (165)	
No	51.1 (90)	5.2 (9)	
Average number of pigmented iris lesions, median (range)	0.5 (0–19.5)	2.5 (0–30)	<0.001
Familiar anamnesis of cutaneous melanoma, % (*n*)			0.541
Yes	2.2 (4)	3.3 (6)	
No	97.8 (178)	96.7 (174)	
Number of melanocytic nevi, mean (SD)			
Diameter 2–5 mm	8.31 (14.39)	28.81 (28.20)	<0.001
Diameter ≥ 5 mm	1.58 (3.4)	4.79 (6.33)	<0.001
Number of melanocytic nevi, median (range)			
Diameter 2–5 mm	4 (0–131)	19.5 (0–194)	<0.001
Diameter ≥ 5 mm	0 (0–25)	3 (0–51)	<0.001

^1^ *n*—the total number of individuals or observations in the sample, ^2^ SD—standard deviation, mm—millimeters in diameter.

**Table 2:** Comparison of the most found iris patterns among subjects with cutaneous melanoma (case group) and controls.

Iris Patterns	Control Group	Case Group	*p*-Value
111 ^1^, % (*n* ^2^)	30.7 (54)	53.9 (97)	<0.001
110 ^3^, % (*n*)	27.8 (49)	2.3 (4)	<0.001
121 ^4^, % (*n*)	5.1 (9)	9.4 (17)	0.097
321 ^5^, % (*n*)	3.4 (6)	7.2 (13)	0.094
221 ^6^, % (*n*)	3.4 (6)	6.7 (12)	0.154

^1^ Blue/grey periphery and blue collaret iris with freckles, ^2^ *n*—the total number of individuals or observations in the sample, ^3^ blue/grey periphery and blue collaret iris without freckles, ^4^ blue/grey periphery and light brown collaret iris with freckles, ^5^ hazel periphery and light brown collaret iris with freckles, ^6^ green periphery and light brown collaret iris with freckles.

**Table 3:** Multivariable binary logistic regression predicting cutaneous melanoma status.

	Odds Ratio	95% Confidence Interval		*p*-Value
		Lower Bound	Upper Bound	
Sex, female (reference)	0.97	0.64	1.49	0.892
Age	1.02	0.99	1.04	0.073
Age groups, ≤50 (reference)				
51–60	1.07	0.60	1.93	0.819
≥61	1.04	0.62	1.74	0.889
Skin color, olive (reference) *				
Fair	0.66	0.27	1.64	0.370
Medium	0.26	0.11	0.62	0.003
Skin type (Fitzpatrick scale), type IV (reference) *				
Type I	1.11	0.54	2.31	0.778
Type II	0.50	0.26	0.95	0.036
Type III	0.67	0.37	1.23	0.197
Hair color, black (reference) *				
Light brown	1.41	0.75	2.67	0.287
Dark brown	0.90	0.45	1.80	0.772
Iris periphery color, dark brown (reference) *				
Blue/grey	2.59	0.49	13.68	0.263
Green	2.76	0.48	15.81	0.255
Hazel	3.73	0.60	23.10	0.157
Light brown	3.02	0.48	19.04	0.239
Iris collaret color, dark brown (reference) *				
Blue	0.61	0.25	1.48	0.278
Light brown	0.59	0.24	1.50	0.269
Presence of pigmented iris lesions *	18.75	8.99	39.12	<0.001
Average number of pigmented iris lesions *	1.13	1.06	1.21	<0.001
Positive familiar anamnesis of cutaneous melanoma *	1.53	0.42	5.55	0.515
Number of melanocytic nevi, median (range) *				
Diameter 2–5 mm	1.07	1.05	1.10	<0.001
Diameter ≥ 5 mm	1.22	1.13	1.32	<0.001

* Odds ratio adjusted by age and sex, mm—millimeters in diameter.

## Data Availability

The datasets used and/or analyzed during the current study are available from the corresponding author on reasonable request.
